# Transitioning subcutaneous immunoglobulin 20% therapies in patients with primary and secondary immunodeficiencies: Canadian real-world study

**DOI:** 10.1186/s13223-022-00709-8

**Published:** 2022-08-07

**Authors:** Paul K. Keith, Juthaporn Cowan, Amin Kanani, Harold Kim, Gina Lacuesta, Jason K. Lee, Jie Chen, Michelle Park, André Gladiator

**Affiliations:** 1 Division of Clinical Immunology and Allergy, Department of Medicine, McMaster University, Hamilton, ON L8N 3Z5 Canada; 2 Division of Infectious Diseases, Department of Medicine; Department of Biochemistry, Microbiology, and Immunology; Centre for Infection, Immunity and Inflammation, University of Ottawa, The Ottawa Hospital Research Institute, Ottawa, ON K1H 8M5 Canada; 3 Division of Allergy and Clinical Immunology, Department of Medicine, St. Paul’s Hospital, University of British Columbia, Vancouver, BC V6Z 1Y6 Canada; 4 Division of Clinical Immunology and Allergy, Department of Medicine, Western University, London, ON N6A 5A5 Canada; 5 Department of Medicine, Dalhousie University, Halifax, NS B3H 2Y9 Canada; 6 Chair of Toronto Allergists and Evidence Based Medical Educator, Toronto, ON M5G 1E2 Canada; 7 Takeda Development Center Americas, Inc., Cambridge, MA 02139 USA; 8 Glattpark-Opfikon , Takeda Pharmaceuticals International AG, Thurgauerstrasse 130, 8152 Zurich, Switzerland

**Keywords:** Ig20Gly, Immunoglobulin replacement therapy, Primary immunodeficiency diseases, Secondary immunodeficiency diseases, Real-world, Subcutaneous immunoglobulin

## Abstract

**Background:**

Real-world data on transitioning to Immune Globulin Subcutaneous (Human) 20% solution (Ig20Gly) are limited. This study aimed to assess infusion parameters and experience of patients with primary (PID) or secondary immunodeficiencies (SID) transitioning to Ig20Gly in clinical practice in Canada.

**Methods:**

Patients with PID or SID who received subcutaneous immunoglobulin (SCIG) for ≥ 3 months before transitioning to Ig20Gly were eligible for this multicenter (n = 6), phase 4, non-interventional, prospective, single-arm study. Ig20Gly infusion parameters, dosing, and adverse events were collected from patient medical records at Ig20Gly initiation and 3, 6, and 12 months post-initiation. Patient satisfaction and quality of life were assessed 12 months post-initiation using validated questionnaires.

**Results:**

The study included 125 patients (PID, n = 60; SID, n = 64; PID + SID, n = 1). Median volume per infusion was 30.0 ml at initiation, and 40.0 ml at 6 and 12 months post-initiation. Most patients administered Ig20Gly weekly and used two infusion sites (primarily abdomen). At each time point, median infusion duration was ≤ 1 h. At 12 months, 61% of infusions were administered via a pump and 39% manually. Headache and infusion-site reactions were the most reported adverse events of interest. Patients expressed overall satisfaction with Ig20Gly at 12 months post-initiation, with all respondents indicating they would like to continue Ig20Gly.

**Conclusions:**

This study provides a detailed description of Ig20Gly infusion parameters, tolerability, and quality of life in clinical practice among patients with PID or SID switching to Ig20Gly from another SCIG and confirms the feasibility of infusing Ig20Gly via pump or manual administration.

*Trial registration* NCT03716700, Registered 31 August 2018, https://clinicaltrials.gov/ct2/show/NCT03716700

**Supplementary Information:**

The online version contains supplementary material available at 10.1186/s13223-022-00709-8.

## Introduction

Primary immunodeficiencies (PID) arise from inborn errors of immunity (more than 430 different genetic conditions identified) and are typically life-long conditions [1]. Secondary immunodeficiencies (SID) are acquired forms of immunodeficiency caused by a broad range of external factors or underlying disease such as cancer, and may be temporary or long-term depending on the etiology [2]. Patients with PID and SID are predisposed to chronic, recurrent, and severe infections, leading to reduced quality of life, and increased morbidity and mortality [2, 3]. Immunoglobulin (IG) replacement therapy (IGRT) is the standard-of-care treatment for patients with PID who have impaired antibody production [4, 5], and is recommended in national guidelines and recent consensus statements [6] for the treatment of SID in patients with recurrent serious bacterial infections or low serum immunoglobulin G (IgG) [6, 7]. IGRT can be administered through an intravenous or subcutaneous route. Subcutaneous immunoglobulin (SCIG) can be self-administered at home, does not require venous access, and has been shown to be as effective as intravenous immunoglobulin (IVIG) at preventing infections in patients with PID, with fewer systemic adverse reactions [8, 9]. However, SCIG infusions require more frequent administration than IVIG (generally ranging from once daily to once every 2 weeks for SCIG vs once every 3–4 weeks by a healthcare professional for IVIG) and a larger number of infusion sites [5, 9].

Immune Globulin Subcutaneous (Human) 20% solution (Ig20Gly [Cuvitru; Baxalta US Inc., a member of the Takeda group of companies, Lexington, MA, USA]) is a highly concentrated IgG formulation stabilized with glycine that can be delivered using smaller total infusion volumes and at higher infusion rates compared with less concentrated SCIG products [10, 11]. Ig20Gly offers patients with immunodeficiencies multiple administration options to customize their IG treatment to fit their needs; these include varying the volume per site, dosing frequency, and infusion rate [10, 12–14]. The safety and efficacy of Ig20Gly have been demonstrated in two phase 2/3 clinical trials (NCT01412385, NCT01218438) in adult and pediatric IG-experienced patients with PID in North America and Europe, with median maximal infusion rates of up to 60 ml/h/site [13, 14]. However, real-world data on Ig20Gly, especially after transitioning from other SCIG therapies, are limited. In April 2018, the Canadian Blood Services (CBS) mandated that patients in Canada (excluding Quebec) transition to Ig20Gly [15]. This provided an opportunity to gain insight on Ig20Gly usage and experience in routine clinical practice across a broad range of patients with PID or SID as they transitioned therapy. The CANadian CUvitru Non-interventional study (CANCUN; NCT03716700) was conducted with the primary objective of assessing Ig20Gly infusion parameters in patients with PID or SID transitioning to Ig20Gly in Canada, under real-life conditions.

## Methods

### Study design

CANCUN was a phase 4, non-interventional, prospective, single-arm study conducted at six centers across Canada (excluding Quebec) between July 1, 2018 and August 31, 2020. Eligible patients were aged older than 2 years, had a documented diagnosis of PID or SID requiring IGRT (as defined by the International Union of Immunological Societies Scientific Committee 2009 and by diagnostic criteria according to Conley et al. 1999) [16], and had received at least 3 months of SCIG therapy prior to receiving Ig20Gly. All patients provided written informed consent.

**Fig. 1:**
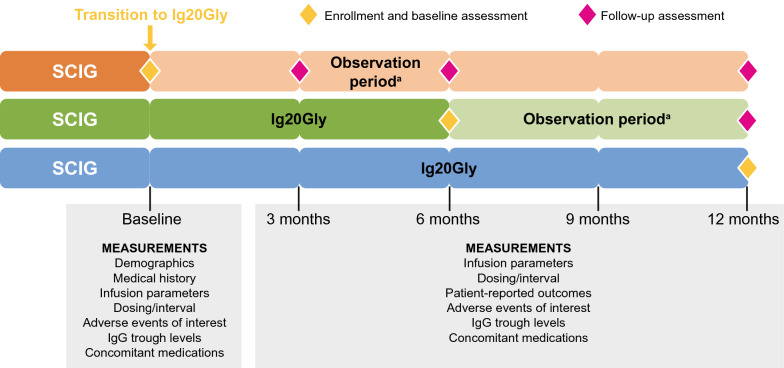
Study design and assessments. ^a^ Time from Ig20Gly initiation. Cohort 1 transitioned to Ig20Gly at study enrollment; Cohort 2 transitioned to Ig20Gly approximately 6 months prior to enrollment; and Cohort 3 transitioned to Ig20Gly approximately 12 months prior to enrollment. Ig20Gly, Immune Globulin Subcutaneous (Human) 20% solution; IgG, immunoglobulin G; SCIG, subcutaneous immunoglobulin

All eligible patients with available data on at least one Ig20Gly administration were included in the analysis and assigned to one of three cohorts based on the time between initiation of Ig20Gly and study enrollment (Fig. 1). Cohort 1 was defined as those patients who transitioned to Ig20Gly at the time of study enrollment (patients who had a dose change within 30 days prior to transition were excluded), with baseline measurements at enrollment and follow-ups at 3 (± 1) months, 6 (± 1) months, and 12 (− 1/ + 2) months. Cohort 2 was defined as those patients who had transitioned to Ig20Gly 6 months (± 2 weeks) prior to study enrollment, with baseline measurements at enrollment and a follow-up at 6 (− 1/ + 2) months (i.e., 12 (− 1/ + 2) months after Ig20Gly initiation). Cohort 3 was defined as those patients who had transitioned to Ig20Gly 12 (− 1/ + 2) months prior to study enrollment, with baseline measurements at enrollment and no follow-up visits.

This study was conducted in accordance with the Declaration of Helsinki. Institutional review board (IRB) approval was obtained centrally (by Advarra), and locally at each site prior to site initiation.

### Study assessments and outcomes

Retrospective and prospective data were collected from patients’ medical records. Patient demographics, medical history, and details of previous SCIG were collected at enrollment (baseline). Data collected at both enrollment and follow-up included infusion parameters, dosing information, and IgG trough levels (Fig. 1). Primary outcomes included infusion volume per site and per infusion, number of infusions per month, number of infusion sites per infusion, and total number of infusions. Information on concomitant infections, including acute serious bacterial infections (defined as bacteremia/sepsis, bacterial meningitis, osteomyelitis/septic arthritis, bacterial pneumonia, and visceral abscesses caused by a recognized bacterial pathogen) were collected at each routine visit. Reports of adverse events (AEs) and serious AEs (SAEs) were collected throughout the observation period. AEs were categorized as ‘AEs of interest for Ig20Gly’ (AEs described as warnings and precautions in the product monograph, AEs reported in previous trials, and AEs observed during post-marketing surveillance) and ‘other’ (not AEs of interest) [13, 14, 17].

Patient-reported health-related quality of life (HRQoL) and treatment satisfaction were assessed using validated, self-administered questionnaires completed by patients at the clinic at 12 months post-Ig20Gly initiation. The Treatment Satisfaction Questionnaire for Medication – 9 items (TSQM-9) [18], the Life Quality Index (LQI) [19, 20], and the Treatment Preference Questionnaire (TPQ) [13] were completed by patients using a study-supplied tablet computer; data were collected into an electronic case report form. These questionnaires were previously used in either one, or both, of the Ig20Gly pivotal studies [13, 14]. In this study, the recall period for the TSQM-9 was 2–3 weeks from the study visit date or since the last Ig20Gly administration. For TSQM-9 and LQI, scores for each domain were calculated by adding up the items in each domain and then transforming into a value ranging from 0 to 100 (higher scores indicated better satisfaction/status). The TPQ was developed to assess patients’ preferences towards the administration of a new SCIG therapy. In this study, patients were asked to respond to 13 questions of the TPQ, which included preference for IG therapy setting (hospital, doctor’s office, home, or other), rating of administration frequency, rating of administration method, and preference for SCIG treatment continuation.

### Analyses

No formal sample size calculation was performed. All analyses are descriptive. Continuous variables were summarized as mean, standard deviation (SD), median, interquartile range (IQR), and minimum and maximum values. For categorical variables, absolute and relative frequency counts were applied. There were no imputations of missing values; the numbers of observations are reported in all analyses.

Analyses were performed for the overall population and for subgroups defined by the mode of Ig20Gly administration (with an infusion pump or without [manual administration]), and by indication (PID or SID).

## Results

### Study population

In total, 126 patients were recruited. Of these, 125 were included in the analysis (one patient did not provide data for at least one Ig20Gly infusion and was excluded from the final analysis). The mean age was 62.1 years (range: 19–83 years) and the majority was female (66.4%). The proportions of IGRT indication were similar between PID (48.8%) and SID (52.0%); one patient had both PID and SID. No pediatric patients were enrolled in this study. Of the 125 patients, 23 (18%) were in Cohort 1, 75 (60%) in Cohort 2, and 27 (22%) in Cohort 3. Patient demographics and baseline clinical characteristics are summarized for the total population and by cohort in Table 1. Prior to initiating Ig20Gly, all patients were receiving Immune Globulin Subcutaneous (Human) 20% liquid, IgPro20 (Hizentra [CSL Behring AG, Bern, Switzerland]). Median (IQR) duration of IgPro20 use was 25.2 (12.3–42.0) months, with the majority of patients administering IgPro20 once weekly (68.8%) and using two or fewer infusion sites (66.7%; n = 68/102). Median maximal infusion rate of IgPro20 was 35.0 ml/h. Overall, 20 patients discontinued the study early: 12 in Cohort 1 and 8 in Cohort 2.

**Table 1:** Patient demographics and baseline clinical characteristics

Parameter^a^	Cohort 1 (n = 23)	Cohort 2 (n = 75)	Cohort 3 (n = 27)	Total (N = 125)
Age, years				
Mean (SD)	65.6 (13.7)	61.8 (13.2)	60.1 (13.9)	62.1 (13.5)
Median (range)	67 (32–82)	65 (19–83)	62 (28–79)	65 (19–83)
Sex				
Female, n (%)	19 (82.6)	47 (62.7)	17 (63.0)	83 (66.4)
Male, n (%)	4 (17.4)	28 (37.3)	10 (37.0)	42 (33.6)
Immunodeficiency diagnosis^b^				
PID diagnosis, n (%)	10 (43.5)	32 (42.7)	19 (70.4)	61 (48.8)
CVID	5 (50.0)	24 (75.0)	14 (73.7)	43 (70.5)
Isolated IG subclass deficiency	0	1 (3.1)	2 (10.5)	3 (4.9)
IgG2 deficiency	0	1 (3.1)	2 (10.5)	3 (4.9)
Specific antibody deficiency with normo- or hypogammaglobulinemia	0	1 (3.1)	1 (5.3)	2 (3.3)
Unclassified antibody deficiency	2 (20.0)	3 (9.4)	0	5 (8.2)
Combined IgA/IgG subclass deficiency	1 (10.0)	0	0	1 (1.6)
Other	2 (20.0)	6 (18.8)	2 (10.5)	10 (16.4)
SID associated with, n (%)	13 (56.5)	44 (58.7)	8 (29.6)	65 (52.0)
Chronic lymphocytic leukemia	3 (23.1)	27 (61.4)	6 (75.0)	36 (55.4)
Multiple myeloma	2 (15.4)	2 (4.5)	0	4 (6.2)
Post-allogeneic HSCT	1 (7.7)	0	0	1 (1.5)
Other	7 (53.8)	15 (34.1)	2 (25.0)	24 (36.9)

^a^n value for each parameter is presented based on available data and percentages are calculated as a proportion of those values

^b^One patient had a diagnosis of both PID and SID

### Infusion and dosing parameters

**Fig. 2:**
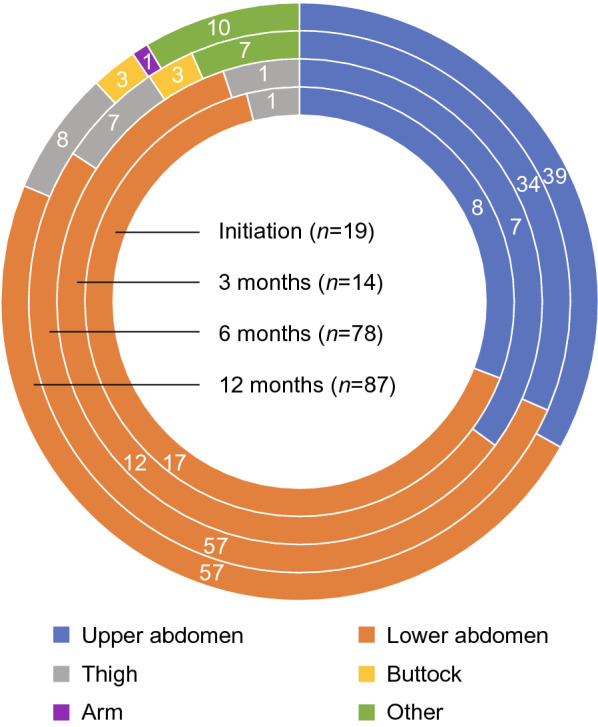
Site of infusion (over the observation period)

In total, 240 infusions were assessed during the study. Infusion and dosing parameters are summarized in Table 2. Across all time points, Ig20Gly was most frequently infused at weekly intervals (≥ 70% of infusions; median of 4 infusions/month). The median volume per infusion was 30.0 ml at initiation and 40.0 ml at months 6 and 12 post-initiation. At each time point, the median infusion duration was ≤ 60 min (IQR: 15–65 min) and interrupted or slowed infusions were rare (1.3%). Patients used a median of two sites per infusion (IQR: 2–4 sites per infusion), most commonly the lower or upper abdomen (Fig. 2). For infusions in the upper and lower abdomen, the median infusion volume per site at 6 months was 25.0 ml and 30.0 ml, respectively, and at 12 months was 30 ml (Additional file 1: Fig. S1). Median weekly Ig20Gly dose was 6.5 g at 3 months and 8.0 g at 6 and 12 months post-initiation. Median monthly Ig20Gly dose by bodyweight was 0.4 g/kg/month at all time points. Median IgG trough levels were 7.8, 11.5, 9.5, and 8.8 g/l at initiation and 3, 6, and 12 months post-initiation, respectively (Table 2).

**Table 2:** Ig20Gly infusion and dosing parameters

	Initiation (n = 23)^a^	Post-initiation		
		3 months (n = 17)^a^	6 months (n = 93)^b^	12 months (n = 107)
Infusion parameters				
Infusion volume/infusion, median (IQR), ml	30 (30–40)	33 (24–45)	40 (25–50)	40 (30–50)
Infusion duration, median (IQR), minutes	35 (15–58)	33 (15–45)	45 (29–63)	60 (30–65)
Number of infusion sites/infusion, median (IQR) 1 site, n (%) 2 sites, n (%) 3 sites, n (%) > 3 sites, n (%)	2 (2–3) 2 (10.5) 11 (57.9) 2 (10.5) 4 (21.1)	2 (2–4) 1 (7.1) 9 (64.3) 0 4 (28.6)	2 (2–3) 10 (12.7) 42 (53.2) 15 (19.0) 12 (15.2)	2 (2–3) 10 (11.5) 49 (56.3) 16 (18.4) 12 (13.8)
Number of infusions/month/patient, median (IQR)	4 (4–4)	4 (4–4)	4 (4–4)	4 (4–4)
Maximal infusion rate/site, median (IQR), ml/h	40.0 (35.0–45.0)	50.9 (50.9–50.9)	43.3 (32.7–58.0)	40.0 (34.0–59.0)
Infusions, n (%), that were:				
Interrupted Slowed Neither slowed nor interrupted Unknown	0 0 12 (52.2) 11 (47.8)	0 0 12 (70.6) 5 (29.4)	0 1 (1.1) 72 (77.4) 20 (21.5)	1 (0.9) 1 (0.9) 76 (71.0) 29 (27.1)
Dosing parameters				
Weekly dose, median (IQR), g	7.5 (6.0–8.0)	6.5 (5.6–8.0)	8.0 (6.0–10.0)	8.0 (6.0–10.0)
Weekly dose per kg, median (IQR), g/kg	0.1 (0.1–0.1)	0.1 (0.1–0.1)	0.1 (0.1–0.1)	0.1 (0.1–0.1)
Dosing interval, n (%)				
Daily 2–6 times/week Once weekly Every 2 weeks Other	0 5 (21.7) 16 (69.6) 2 (8.7) 0	0 2 (11.8) 13 (76.5) 2 (11.8) 0	1 (1.1) 19 (20.4) 65 (69.9) 7 (7.5) 1 (1.1)	0 19 (17.9) 81 (76.4) 4 (3.8) 2 (1.9)
IgG trough levels, g/l^c^				
Median (IQR) Range	7.8 (7.4–10.7)^d^ 0.8–12.1	11.5 (4.7–11.6) 4.7–11.6	9.5 (7.9–10.7) 5.0–14.4	8.8 (8.0–10.9) 3.6–14.5

n is number of patients. Total n for each time point is based on available data

^a^ Cohort 1 only

^b^ Cohorts 1 and 2 only

^c^ Available data: initiation, n = 9; 3 months, n = 3; 6 months, n = 46; 12 months, n = 59

^d^ All patients were SCIG-experienced at initiation; this value is the result of prior SCIG treatment during the transition to Ig20Gly

### Safety

In total, 20 AEs of interest (in 15 patients) and 29 other AEs (in 20 patients) were reported (Table 3). Most AEs were mild or moderate in severity. There were five SAEs reported by five patients (stroke, post-chemotherapy infection, neck/throat abscess, pneumonia, febrile neutropenia; one case each); one (stroke) was considered as possibly related to Ig20Gly. Headache and infusion-site reactions were the most frequently reported AEs of interest (4.8% and 4.0% of all patients, respectively; Additional file 2: Table S1). There were no deaths during the study.

**Table 3:** Summary of AEs

	AEs of interest		Other AEs		SAEs^a^	
	Patients, n (%) (N = 125)	AEs, n	Patients, n (%) (N = 125)	AEs, n	Patients n (%) (N = 125)	AEs, n
Any AE	15 (12.0)	20	20 (16.0)	29	5 (4.0)	5
Severity of AE^b^						
Mild	10 (8.0)	13	11 (8.8)	14	0	0
Moderate	5 (4.0)	6	6 (4.8)	12	1 (0.8)	1
Severe	1 (0.8)	1	3 (2.4)	3	4 (3.2)	4
AEs considered related to Ig20Gly						
Related	7 (5.6)	8	3 (2.4)	4	0	0
Possibly related	5 (4.0)	5	4 (3.2)	6	1 (0.8)	1
Probably related	2 (1.6)	2	2 (1.6)	2	0	0

^a^ SAEs were defined as any untoward medical occurrence that at any dose results in death, is life-threatening, requires inpatient hospitalization or prolongation of existing hospitalization, results in persistent or significant disability/incapacity, is a congenital abnormality/birth defect, or is an important medical event

^b^ AE severity was defined as follows: mild – usually transient and may require only minimal treatment or therapeutic intervention (the event does not generally interfere with usual activities of daily living); moderate – usually alleviated with specific therapeutic intervention (the event interferes with usual activities of daily living, causing discomfort, but poses no significant or permanent risk of harm to the patient); severe – interrupts usual activities of daily living, or significantly affects clinical status, or may require intensive therapeutic intervention

### Concomitant infections

Bacterial infections were reported by 22.6% of patients at 6 months (Cohorts 1 and 2 only) and 19.8% of patients at 12 months post-initiation. Of these, three infections at 6 months (two in the PID subgroup and one in the SID subgroup) and three infections at 12 months (all in the SID subgroup) were recorded by the physician as acute serious bacterial infections (all pneumonia).

### Patient-reported health-related quality of life outcomes

TSQM-9, LQI, and TPQ were completed by 103, 100, and 101 patients, respectively, and indicated a positive patient impression of the switch to Ig20Gly. For TSQM-9 (scale 0–100), mean (SD) domain scores were 79.4 (19.8) for global satisfaction, 78.0 (19.9) for effectiveness, and 75.2 (15.9) for convenience of Ig20Gly use. For LQI (scale 0–100), mean (SD) domain scores were 92.4 (9.0) for treatment interference, 85.1 (13.4) for therapy-related problems, 95.5 (7.8) for therapy setting, and 92.7 (11.2) for treatment costs.

**Fig. 3:**
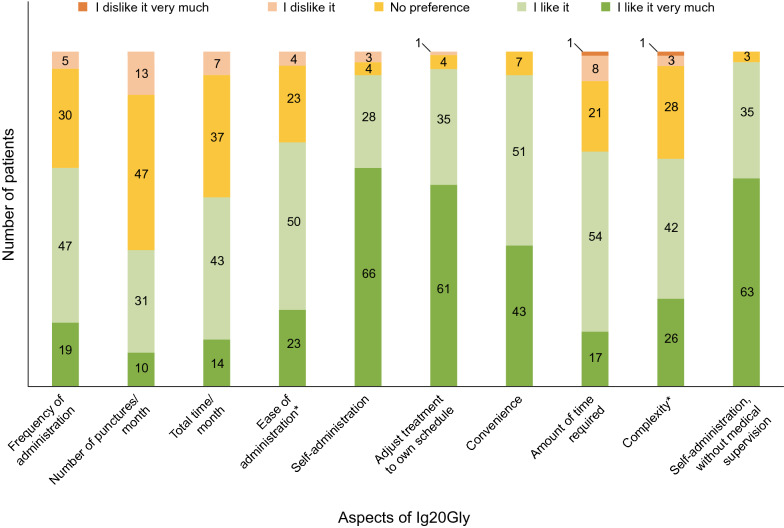
Patient satisfaction with aspects of Ig20Gly administration (assessed using the Treatment Preference Questionnaire). n = 101 patients. ^a^ n = 100 patients. Ig20Gly, Immune Globulin Subcutaneous (Human) 20% solution

Overall, 96.0% of TPQ respondents preferred at-home infusions. All patients expressed an interest in continuing Ig20Gly treatment. Patient satisfaction with individual aspects of Ig20Gly administration (assessed by TPQ) are summarized in Fig. 3; aspects relating to the ability to adjust treatment to own schedule, self-administration (in general and without medical supervision), and convenience were the highest rated (≥ 93% of respondents rating as ‘I like it’ or ‘I like it very much’).

### Subgroup analysis by mode of administration at 12 months

Overall, 43% (n = 54) of the study population administered Ig20Gly manually. Infusion and dosing parameters by mode of administration (infusion pump vs manual) at 12 months are summarized in Table 4 (full details are reported for all time points in Additional file 2: Table S2). The upper and lower abdomen were the most common infusion sites, irrespective of the mode of administration. Compared with patients using a pump, patients using manual administration did so at a lower median (IQR) infusion volume per infusion (30 [20–40] ml vs 43 [40–60] ml) and shorter infusion duration (24 [10–40] vs 60 [45–73] minutes), were more likely to use two or fewer infusion sites (96.3% vs 55.0% of the time), and tended to infuse at more frequent dosing intervals (Table 4). Despite these differences in infusion parameters, IgG trough levels were similar for the manual push and infusion pump cohorts at 6 and 12 months (Additional file 2: Table S2). TSQM-9, LQI, and TPQ results were comparable between patients who administered Ig20Gly with an infusion pump and patients who administered manually (Additional file 2: Table S3). Safety analysis by mode of administration is provided in Additional file 2: Table S4.

**Table 4:** Subgroup analysis by mode of administration and indication for IGRT

	Mode of administration		Indication for IGRT	
	Infusion pump	Manual	PID	SID
Patient characteristics at baseline	n = 71	n = 54	n = 61^a^	n = 64^b^
Age, median (range)	66 (28–83)	63 (19–82)	57 (19–79)	69 (48–83)
Sex, n (%)				
Female	44 (62.0)	39 (72.2)	47 (77.0)	36 (56.3)
Male	27 (38.0)	15 (27.8)	14 (23.0)	28 (43.8)
Mode of administration, n (%)				
Infusion pump	71 (100)	0	33 (54.1)	38 (59.4)
Manual	0	54 (100)	28 (45.9)	26 (40.6)
Indication for IGRT, n (%)				
PID^a^	33 (46.5)	28 (51.9)	61 (100)	0
SID^b^	38 (53.5)	26 (48.1)	0	64 (100)
Infusion and dosing parameters at 12 months^c^	n = 65	n = 42^d^	n = 52	n = 55
Infusion volume/infusion, median (IQR), ml	43 (40–60)	30 (20–40)	40 (25–50)	40 (35–50)
Infusion duration, median (IQR), minutes	60 (45–73)	24 (10–40)	43 (20–65)	60 (40–65)
Number of infusion sites/infusion, median (IQR)	2 (2–3)	2 (1–2)	2 (2–4)	2 (2–3)
1 site, n (%)	1 (1.7)	9 (33.3)	8 (18.6)	2 (4.5)
2 sites, n (%)	32 (53.3)	17 (63.0)	21 (48.8)	28 (63.6)
3 sites, n (%)	16 (26.7)	0	3 (7.0)	13 (29.5)
> 3 sites, n (%)	11 (18.3)	1 (3.7)	11 (25.6)	1 (2.3)
Number of infusions/month/patient, median (IQR)	4 (4–4)	4 (4–8)	4 (4–8)	4 (4–4)
Maximal infusion rate/site, median (IQR), (ml/h)^e^	40.0 (34.0–59.0)	–	33.5 (25.7–40.0)	40.0 (35.0–60.0)
Weekly dose, median (IQR), g	8.0 (6.0–10.0)	8.0 (6.0–8.0)	9.0 (7.0–12.0)	8.0 (6.0–10.0)
Dosing interval, *n* (%)				
Daily	0	0	0	0
2–6 times/week	2 (3.1)	17 (40.5)	15 (28.8)	4 (7.4)
Once weekly	56 (87.5)	25 (59.5)	34 (65.4)	47 (87.0)
Every 2 weeks	4 (6.3)	–	2 (3.8)	2 (3.7)
Other	2 (3.1)	–	1 (1.9)	1 (1.9)
IgG trough levels, g/l				
Median (IQR)	8.6 (7.9–10.6)	9.1 (8.3–11.0)	9.9 (8.6–11.8)	8.3 (7.9–9.0)
Range	3.6–14.5	5.6–13.4	3.6–13.4	5.6–14.5

^a^ Includes one patient with PID and SID

^b^ SID only

^c^ n value for each parameter is presented based on available data and percentages are calculated as a proportion of those values

^d^ One patient in this group used an infusion pump at 12 months

^e^ Maximal infusion rate per site can only be analyzed for patients using pump administration and is the same for all sites in that infusion

### Subgroup analysis by indication at 12 months

Compared with the SID only subgroup (n = 64), the PID subgroup (n = 61) tended to be younger, contain a higher proportion of females, and were slightly more likely to infuse Ig20Gly manually (Table 4). Infusion and dosing parameters by indication at 12 months are summarized in Table 4 (full details are reported for all the time points in Additional file 2: Table S5). The median (IQR) infusion duration was 43 (20–65) minutes for patients with PID and 60 (40–65) minutes for patients with SID. All other infusion parameters were generally similar for the two subgroups. Median IgG trough levels at 12 months were 9.9 and 8.3 g/l for patients with PID and SID, respectively. TSQM-9, LQI, and TPQ score were comparable between patients with PID and SID (Additional file 2: Table S3). Safety analysis by indication is provided in Additional file 2: Table S6.

## Discussion

IGRT is typically a life-long treatment for PID and may be temporary or life-long in patients with SID. Consequently, it is important to supplement the clinical evidence from pivotal studies with real-world evidence from studies designed to explore how patients are using these therapies to match not only their clinical needs, but also the needs of their day-to-day schedules, as well as their experience and quality of life while receiving these treatments. CANCUN provides real-world insights into Ig20Gly infusion parameters, tolerability, and HRQoL outcomes in a broad range of adults with PID or SID transitioning from another 20% SCIG therapy in Canada.

Median infusion volume per infusion reached 40 ml at 6 and 12 months post-initiation, and across all time points, median infusion duration was at most 1 h. Most patients infused into the upper or lower abdomen and used two or fewer infusion sites per infusion. While Ig20Gly was most frequently infused at weekly intervals (≥ 70% of infusions), in approximately 10–20% of infusions, patients chose to infuse at more frequent intervals. Furthermore, compared with SCIG therapy used prior to Ig20Gly initiation, patients in this study infused Ig20Gly at higher median maximal infusion rates at all time points assessed and had decreased infusion duration at all time points except 12 months.

Ig20Gly infusion parameters outside of a clinical trial setting have also recently been explored in a retrospective analysis of records of patients participating in the HelloCuvitru program, a patient support program in the United States providing Ig20Gly free of charge for the first four infusions to patients aged ≥ 2 years with PID and no prior experience with Ig20Gly. Consistent with CANCUN, HelloCuvitru found that most patients (83%) received Ig20Gly at two or fewer infusion sites and reported a median infusion duration of less than 1 h. Median infusion volume per site tended to be somewhat higher in HelloCuvitru than in the current study (40 ml vs 30 ml, typically, in CANCUN), and while weekly administration remained the most common treatment schedule (as observed in CANCUN), almost 50% of the adult population within HelloCuvitru infused at a less frequent schedule (between 8 and 14 days) [21]. The differences seen between our study and HelloCuvitru may be due to patient population, treatment setting (i.e., free trial program), and study design. First, the current study included patients who administered Ig20Gly manually and patients with SID; groups which were not included in HelloCuvitru. Both populations tend to administer Ig20Gly at an overall lower median infusion volume per site than individuals with PID using an infusion pump. Additionally, HelloCuvitru was a program that offered four free doses of Ig20Gly, which patients may have chosen to receive at more extended intervals relative to the current study [21]. The infusion parameters observed in CANCUN were also similar to those of the European and North American pivotal trials of Ig20Gly, with regard to infusion duration and number of infusion sites [13, 14]. However, compared with CANCUN, the European trial reported a lower median infusion volume per site (16.6 ml/site) and lower median maximum infusion rate per site (20 ml/site/h vs 40–50 ml/site/h) [13]. This may, in part, be related to the much lower median age (17 years) and lower median weight (63 kg) in the European pivotal trial compared with patients in CANCUN (62–67 years and 65–75 kg, respectively) [13]. Finally, the infusion protocols of the Ig20Gly pivotal trials required patients to receive infusions every 7 days [13, 14], whereas both the HelloCuvitru program and the CANCUN study allowed for more flexibility in infusion schedules [21]. Despite some differences from the pivotal trials [13, 14] and HelloCuvitru [21], the findings in this study suggest that the higher infusion rates, shorter infusion times, and fewer infusion sites relative to other licensed SCIG products can translate to real-world settings.

The safety and AE profile in the real-world setting of this study was consistent with that observed in the controlled setting of the pivotal trials, with local infusion-site reactions and headache being the most reported AEs of interest [13, 14]. There was one SAE (stroke) reported that was considered as possibly related to Ig20Gly.

Patient-reported HRQoL outcomes at 12 months post-initiation, which included the TSQM-9, LQI, and TPQ questionnaires, showed that both the PID and SID cohorts had a favorable impression of Ig20Gly usage and expressed interest in continuing Ig20Gly treatment, irrespective of mode of administration. Overall, 96% of patients preferred home-based infusions and almost all liked the option of self-administration without medical supervision. Data from the two Ig20Gly pivotal PID trials have shown that patients experienced significantly improved satisfaction with treatment after transitioning to Ig20Gly [13, 14].

Subgroup analysis of infusion parameters by mode of administration provided new evidence on administration of Ig20Gly without an infusion pump and confirmed the feasibility of infusing manually. Patients using manual administration had a shorter total median duration of infusion, lower median infusion volume per infusion, and were more likely to use two or fewer sites than patients using an infusion pump but tended to infuse more frequently. Despite this, IgG trough levels were generally similar for manual administration and infusion pump cohorts. The sites most used for infusion were similar for the two groups. Previous studies on manual SCIG administration, also known as rapid- or manual-push SCIG administration, reported that this method was an effective and well-tolerated alternative to SCIG administration via an infusion pump in pediatric [22], adult [23], and obese patients [24]. Another study found that some patients preferred manual SCIG administration rather than using an infusion pump because of the convenience and reduction in infusion time [25].

CANCUN also provides evidence on the feasibility of using Ig20Gly in patients with SID. Infusion parameters were similar for patients with PID and SID, except for median infusion duration, which tended to be slightly longer in patients with SID. A recent systematic literature review of clinical studies of IGRT in SID associated with hematological malignancies and stem-cell transplants found several beneficial effects of IGRT on clinical outcomes and quality of life, although acknowledging a paucity of clinical trial data post-1990s in this area [7]. The review reported all-cause mortality of 11.1–60.0% (across six studies), and infection-related mortality of 0–14.3% (across eight studies) [7]. In the present study, acute serious bacterial infections were reported in one patient with SID at 6 months and in three patients with SID at 12 months post-initiation; notably, no deaths occurred in any patient group over the course of the study. It has been shown that infection rate is inversely correlated with IgG trough levels following SCIG administration in patients with primary antibody deficiency [26]. At 12 months, median IgG trough levels were slightly lower in patients with SID vs PID (8.3 vs 9.9 g/l), although this is still higher than the level of ≥ 5 g/l that IGRT therapies aim to maintain to provide adequate protection from infection [27, 28], and higher than the level of > 8 g/l that has the potential to improve pulmonary outcomes (albeit the range of values in these studies showed that some participants had trough levels < 5 g/l and this may have been due to dose administered, protein-losing enteropathy, rapid metabolism or adherence) [27, 28]. Further, our results show that HRQoL outcomes with Ig20Gly are comparable in patients with PID and SID.

There are several limitations to consider when interpreting the present findings. These include the inherent biases of retrospective real-world chart review studies together with their limitations and potential for missing data. In addition, Ig20Gly infusion parameters in this study reflect routine clinical practice in Canada (excluding Quebec) and may not be widely generalizable owing to differences in clinical practice and patient characteristics. While the protocol allowed for the inclusion of patients older than 2 years, no pediatric patients were included. This may reflect a lack of pediatrics sites enrolled and/or targeting adults first during the transition to Ig20Gly from IgPro20. A direct comparison to other IG products is limited to information on prior IgPro20 use collected at initiation. In September 2018, certain IG products were phased out from the CBS formulary, thereby mandating transition from previous SCIG 20% therapy to Ig20Gly between April and September 2018 [29]. This transition period allowed each patient to be trained and initiated on Ig20Gly, however this mandate did not allow patients to choose their SCIG therapy.

## Conclusion

This study provides a detailed description of Ig20Gly infusion parameters, tolerability, and HRQoL in clinical practice among patients with PID or SID switching to Ig20Gly from another SCIG product, and confirms the feasibility of infusing the product either manually or by using a pump. The findings of this real-world study are broadly consistent with previous findings from the Ig20Gly pivotal trials.

## Supplementary Information

**Additional file 1: Fig. S1.** Infusion volume per site (at 6 and 12 months).

**Additional file 2: **Additional tables.

## Data Availability

The data sets, including the redacted study protocol, redacted statistical analysis plan, and individual participant data supporting the results reported in this article, will be made available within 3 months from initial request, to researchers who provide a methodologically sound proposal. The data will be provided after its de-identification, in compliance with applicable privacy laws, data protection and requirements for consent and anonymization.

## Abbreviations

AE: Adverse event
CANCUN: CANadian CUvitru Non-interventional
CBS: Canadian Blood Services
CVID: Common variable immunodeficiency
HRQoL: Health-related quality of life
HSCT: Hematopoietic stem-cell transplantation
IG: Immunoglobulin
Ig20Gly: Immune Globulin Subcutaneous (Human) 20% solution
IgA: Immunoglobulin A
IgG: Immunoglobulin G
IgPro20: Immune Globulin Subcutaneous (Human) 20% liquid
IGRT: Immunoglobulin replacement therapy
IQR: Interquartile range
IRB: Institutional review board
IVIG: Intravenous immunoglobulin
LQI: Life Quality Index
PID: Primary immunodeficiencies
SAE: Serious adverse event
SCIG: Subcutaneous immunoglobulin
SD: Standard deviation
SID: Secondary immunodeficiencies
TPQ: Treatment Preference Questionnaire
TSQM-9: Treatment Satisfaction Questionnaire for Medication-9 items
