# Correction: Transitioning subcutaneous immunoglobulin 20% therapies in patients with primary and secondary immunodeficiencies: Canadian real‑world study

**DOI:** 10.1186/s13223-023-00805-3

**Published:** 2023-06-23

**Authors:** Paul K. Keith, Juthaporn Cowan, Amin Kanani, Harold Kim, Gina Lacuesta, Jason K. Lee, Jie Chen, Michelle Park, André Gladiator

**Affiliations:** 1 Division of Clinical Immunology and Allergy, Department of Medicine, McMaster University, Hamilton, ON L8N 3Z5 Canada; 2 Division of Infectious Diseases, Department of Medicine, Department of Biochemistry, Microbiology, and Immunology; Centre for Infection, Immunity and Inflammation, University of Ottawa, The Ottawa Hospital Research Institute, Ottawa, ON K1H 8M5 Canada; 3 Division of Allergy and Clinical Immunology, Department of Medicine, St. Paul’s Hospital, University of British Columbia, Vancouver, BC V6Z 1Y6 Canada; 4 Division of Clinical Immunology and Allergy, Department of Medicine, Western University, London, ON N6A 5A5 Canada; 5 Department of Medicine, Dalhousie University, Halifax, NS B3H 2Y9 Canada; 6 Chair of Toronto Allergists and Evidence Based Medical Educator, Toronto, ON M5G 1E2 Canada; 7 Takeda Development Center Americas, Inc., Cambridge, MA 02139 USA; 8 Glattpark‑Opfikon, Takeda Pharmaceuticals International AG, Thurgauerstrasse 130, 8152 Zurich, Switzerland

**Correction: Allergy, Asthma & Clinical Immunology (2022) 18:70** 10.1186/s13223-022-00709-8

Following the publication of our article [1], a previously undetected error in the data set has come to our attention. MedDRA-coded preferred terms (PTs) were not coded in the derived adverse event (AE) data set used for the summary of AEs and AEs of interest. While the overall number of AEs was reported correctly in the published article, three AEs that were originally categorized as ‘other’ have now been re-categorized. Consequently, safety data presented in the Results require correction.

Firstly, in the *Safety* subsection, the first sentence stated: “In total, 20 AEs of interest (in 15 patients) and 29 other AEs (in 20 patients) were reported (Table 3).”

It should instead read: “In total, 23 AEs of interest (in 16 patients) and 26 other AEs (in 19 patients) were reported (Table 3).”

The referenced *Table 3 (Summary of AEs)* originally presented the following data:

**Table:** 

	AEs of interest		Other AEs		SAEs^a^	
	Patients, n (%) (N = 125)	AEs, n	Patients, n (%) (N = 125)	AEs, n	Patients, n (%) (N = 125)	AEs, n
Any AE	15 (12.0)	20	20 (16.0)	29	5 (4.0)	5
Severity of AE^b^						
Mild	10 (8.0)	13	11 (8.8)	14	0	0
Moderate	5 (4.0)	6	6 (4.8)	12	1 (0.8)	1
Severe	1 (0.8)	1	3 (2.4)	3	4 (3.2)	4
AEs considered related to Ig20Gly						
Related	7 (5.6)	8	3 (2.4)	4	0	0
Possibly related	5 (4.0)	5	4 (3.2)	6	1 (0.8)	1
Probably related	2 (1.6)	2	2 (1.6)	2	0	0

*Table 3* should instead present the following corrected data:

**Table:** 

	AEs of interest		Other AEs		SAEs^a^	
	Patients, n (%) (N = 125)	AEs, n	Patients, n (%) (N = 125)	AEs, n	Patients, n (%) (N = 125)	AEs, n
Any AE	16 (12.8)	23	19 (15.2)	26	5 (4.0)	5
Severity of AE^b^						
Mild	11 (8.8)	14	10 (8.0)	13	0	0
Moderate	5 (4.0)	8	6 (4.8)	10	1 (0.8)	1
Severe	1 (0.8)	1	3 (2.4)	3	4 (3.2)	4
AEs considered related to Ig20Gly						
Related	7 (5.6)	8	3 (2.4)	4	0	0
Possibly related	5 (4.0)	7	4 (3.2)	4	1 (0.8)	1
Probably related	2 (1.6)	2	2 (1.6)	2	0	0

Safety data in *Additional file 2: Table S1 (AEs of interest)*, referenced in the *Safety* subsection, were originally presented as follows:

**Table:** 

AE of interest	Patients, n (%) (N = 125)	
	All causality	Considered related to Ig20Gly
Nausea	2 (1.6)	1 (0.8)
Diarrhea	1 (0.8)	0
Headache	6 (4.8)	3 (2.4)
Cough	2 (1.6)	0
Stroke	1 (0.8)	0
Fatigue	2 (1.6)	0
Infusion-site erythema (redness)	1 (0.8)	1 (0.8)
Infusion-site pain	3 (2.4)	3 (2.4)
Infusion-site pruritus (itchiness)	1 (0.8)	0

*Table S1* should instead present the following corrected data for all causality fatigue:

**Table:** 

AE of interest	Patients, n (%) (N = 125)	
	All causality	Considered related to Ig20Gly
Nausea	2 (1.6)	1 (0.8)
Diarrhea	1 (0.8)	0
Headache	6 (4.8)	3 (2.4)
Cough	2 (1.6)	0
Stroke	1 (0.8)	0
Fatigue	3 (2.4)	0
Infusion-site erythema (redness)	1 (0.8)	1 (0.8)
Infusion-site pain	3 (2.4)	3 (2.4)
Infusion-site pruritus (itchiness)	1 (0.8)	0

Safety data in *Additional file 2: Table S4 (AEs by mode of Ig20Gly administration)*, referenced in the *Subgroup analysis by mode of administration at 12 months* subsection, were originally presented as follows:

**Table:** 

	AEs of interest				Other AEs				SAEs			
	Infusion pump		Manual		Infusion pump		Manual		Infusion pump		Manual	
	Patients, n (%) (N = 71)	AEs, n	Patients, n (%) (N = 54)	AEs, n	Patients, n (%) (N = 71)	AEs, n	Patients, n (%) (N = 54)	AEs, n	Patients, n (%) (N = 71)	AEs, n	Patients, n (%) (N = 54)	AEs, n
Any AE	6 (8.5)	9	9 (16.7)	11	12 (16.9)	13	8 (14.8)	16	5 (7.0)	5	0	0
Severity of AE												
Mild	4 (5.6)	7	6 (11.1)	6	6 (8.5)	7	5 (9.3)	7	0	0	0	0
Moderate	1 (1.4)	1	4 (7.4)	5	3 (4.2)	3	3 (5.6)	9	1 (1.4)	1	0	0
Severe	1 (1.4)	1	0	0	3 (4.2)	3	0	0	4 (5.6)	4	0	0
AEs considered related to Ig20Gly												
Related	3 (4.2)	3	4 (7.4)	5	2 (2.8)	3	1 (1.9)	1	0	0	0	0
Possibly related	2 (2.8)	2	3 (5.6)	3	2 (2.8)	2	2 (3.7)	4	1 (1.4)	1	0	0
Probably related	1 (1.4)	1	1 (1.9)	1	0	0	2 (3.7)	2	0	0	0	0

*Table S4* should instead present the following corrected data for the manual administration subgroup:

**Table:** 

	AEs of interest				Other AEs				SAEs			
	Infusion pump		Manual		Infusion pump		Manual		Infusion pump		Manual	
	Patients, n (%) (N = 71)	AEs, n	Patients, n (%) (N = 54)	AEs, n	Patients, n (%) (N = 71)	AEs, n	Patients, n (%) (N = 54)	AEs, n	Patients, n (%) (N = 71)	AEs, n	Patients, n (%) (N = 54)	AEs, n
Any AE	6 (8.5)	9	10 (18.5)	14	12 (16.9)	13	7 (13.0)	13	5 (7.0)	5	0	0
Severity of AE												
Mild	4 (5.6)	7	7 (13.0)	7	6 (8.5)	7	4 (7.4)	6	0	0	0	0
Moderate	1 (1.4)	1	4 (7.4)	7	3 (4.2)	3	3 (5.6)	7	1 (1.4)	1	0	0
Severe	1 (1.4)	1	0	0	3 (4.2)	3	0	0	4 (5.6)	4	0	0
AEs considered related to Ig20Gly												
Related	3 (4.2)	3	4 (7.4)	5	2 (2.8)	3	1 (1.9)	1	0	0	0	0
Possibly related	2 (2.8)	2	3 (5.6)	5	2 (2.8)	2	2 (3.7)	2	1 (1.4)	1	0	0
Probably related	1 (1.4)	1	1 (1.9)	1	0	0	2 (3.7)	2	0	0	0	0

Finally, safety data in *Additional file 2: Table S6 (AEs by indication)*, referenced in the *Subgroup analysis by indication at 12 months* subsection, were originally presented as follows:

**Table:** 

Parameter, n (%)	AEs of interest				Other AEs				SAEs			
	PID		SID		PID		SID		PID		SID	
	Patients, n (%) (N = 61)	AEs, n	Patients, n (%) (N = 64)	AEs, n	Patients, n (%) (N = 61)	AEs, n	Patients, n (%) (N = 64)	AEs, n	Patients, n (%) (N = 61)	AEs, n	Patients, n (%) (N = 64)	AEs, n
Any AE	10 (16.4)	15	5 (7.8)	5	8 (13.1)	14	12 (18.8)	15	1 (1.6)	1	4 (6.3)	4
Severity of AE												
Mild	7 (11.5)	10	3 (4.7)	3	6 (9.8)	8	5 (7.8)	6	0	0	0	0
Moderate	3 (4.9)	4	2 (3.1)	2	2 (3.3)	6	4 (6.3)	6	0	0	1 (1.6)	1
Severe	1 (1.6)	1	0	0	0	0	3 (4.7)	3	1 (1.6)	1	3 (4.7)	3
AEs considered related to Ig20Gly												
Related	5 (8.2)	6	2 (3.1)	2	2 (3.3)	2	1 (1.6)	2	0	0	0	0
Possibly related	3 (4.9)	3	2 (3.1)	2	2 (3.3)	2	2 (3.1)	4	1 (1.6)	1	0	0
Probably related	1 (1.6)	1	1 (1.6)	1	1 (1.6)	1	1 (1.6)	1	0	0	0	0

*Table﻿ S6* should instead present the following corrected data:

**Table:** 

Parameter, n (%)	AEs of interest				Other AEs				SAEs			
	PID		SID		PID		SID		PID		SID	
	Patients, n (%) (N = 61)	AEs, n	Patients, n (%) (N = 64)	AEs, n	Patients, n (%) (N = 61)	AEs, n	Patients, n (%) (N = 64)	AEs, n	Patients, n (%) (N = 61)	AEs, n	Patients, n (%) (N = 64)	AEs, n
Any AE	11 (18.0)	16	5 (7.8)	7	7 (11.5)	13	12 (18.8)	13	1 (1.6)	1	4 (6.3)	4
Severity of AE												
Mild	8 (13.1)	11	3 (4.7)	3	5 (8.2)	7	5 (7.8)	6	0	0	0	0
Moderate	3 (4.9)	4	2 (3.1)	4	2 (3.3)	6	4 (6.3)	4	0	0	1 (1.6)	1
Severe	1 (1.6)	1	0	0	0	0	3 (4.7)	3	1 (1.6)	1	3 (4.7)	3
AEs considered related to Ig20Gly												
Related	5 (8.2)	6	2 (3.1)	2	2 (3.3)	2	1 (1.6)	2	0	0	0	0
Possibly related	3 (4.9)	3	2 (3.1)	4	2 (3.3)	2	2 (3.1)	2	1 (1.6)	1	0	0
Probably related	1 (1.6)	1	1 (1.6)	1	1 (1.6)	1	1 (1.6)	1	0	0	0	0

Please note that the conclusions made in the original publication are unaffected by the errors identified.
