# Supplementary figures and images for: Transitioning subcutaneous immunoglobulin 20% therapies in patients with primary and secondary immunodeficiencies: Canadian real-world study
Source: Allergy Asthma Clin Immunol. 2022 Aug 7;18:70. doi: 10.1186/s13223-022-00709-8 (PMC9358831; doi:10.1186/s13223-022-00709-8)

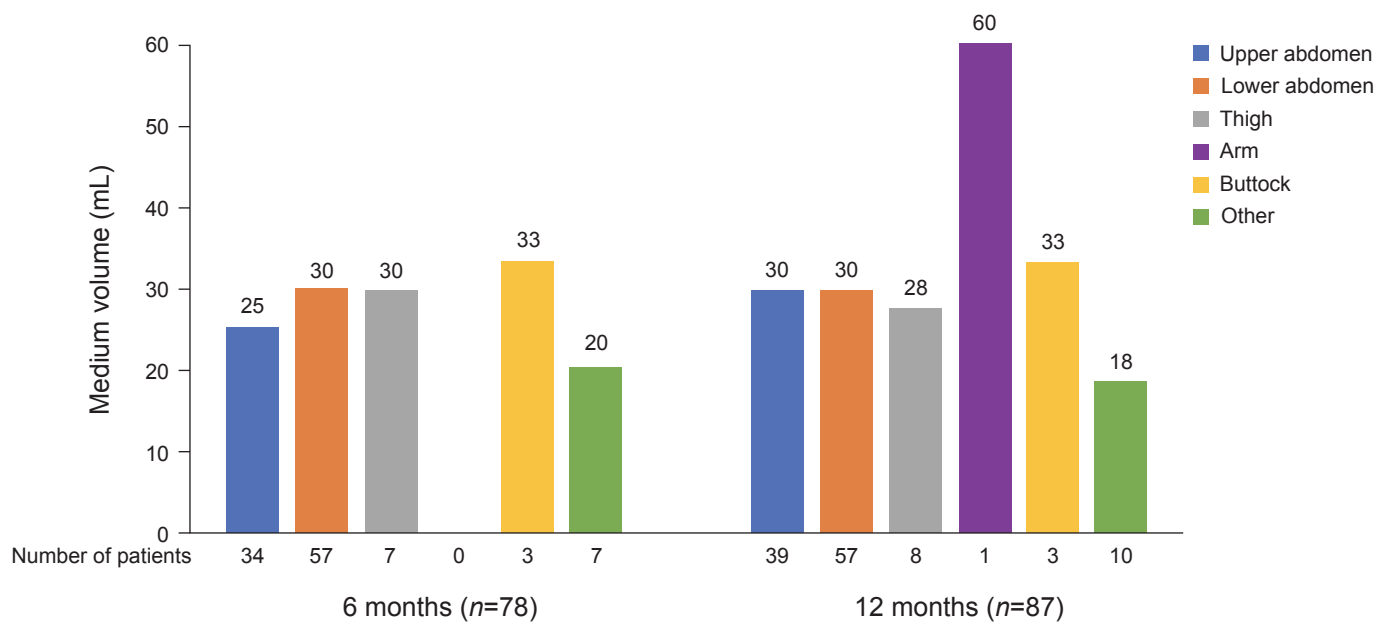

Supplement: Supplementary file 1 — Additional file 1: Fig. S1. Infusion volume per site (at 6 and 12 months). [file 13223_2022_709_MOESM1_ESM.pdf]
