# Supplementary material for: Transitioning subcutaneous immunoglobulin 20% therapies in patients with primary and secondary immunodeficiencies: Canadian real-world study
Source: Allergy Asthma Clin Immunol. 2022 Aug 7;18:70. doi: 10.1186/s13223-022-00709-8 (PMC9358831; doi:10.1186/s13223-022-00709-8)
Supplement: Supplementary file 2 — Additional file 2: Additional tables. [file 13223_2022_709_MOESM2_ESM.docx]

# Additional file 2

## Table S1. AEs of interest

|  | **Patients, n (%)**  **(N=125)** | |
| --- | --- | --- |
| **AE of interest** | **All causality** | **Considered related to Ig20Gly** |
| Nausea | 2 (1.6) | 1 (0.8) |
| Diarrhea | 1 (0.8) | 0 |
| Headache | 6 (4.8) | 3 (2.4) |
| Cough | 2 (1.6) | 0 |
| Stroke | 1 (0.8) | 0 |
| Fatigue | 2 (1.6) | 0 |
| Infusion-site erythema (redness) | 1 (0.8) | 1 (0.8) |
| Infusion-site pain | 3 (2.4) | 3 (2.4) |
| Infusion-site pruritus (itchiness) | 1 (0.8) | 0 |

## Table S2. Infusion and dosing parameters by mode of Ig20Gly administration

|  | **Initiation**^a^ | | **Post-initiation** | | | | | | | | | | |
| --- | --- | --- | --- | --- | --- | --- | --- | --- | --- | --- | --- | --- | --- |
|  |  |  | **3 months**^a^ | | | **6 months**^b^ | | | | **12 months** | | | |
|  | **Infusion pump**  **(n=6)** | **Manual**  **(n=17)** | **Infusion pump**  **(n=5)** | **Manual**  **(n=12)** | | **Infusion pump**  **(n=53)** | | | **Manual**  **(n=40)** | **Infusion pump**  **(n=65)** | | **Manual**  **(n=42)** | |
| **Infusion parameters** | | | | | | | | | | | | | |
| Infusion volume/infusion, median (IQR), ml | 53 (40–60) | 30 (25–38) | 55 (50–70) | | 30 (20–40) | | 50 (40–60) | 30 (20–40) | | | 43 (40–60) | | 30 (20–40) |
| Infusion duration, median (IQR), minutes | 60 (45–60) | 15 (10–30) | 50 (37–68) | | 15 (10–15) | | 55 (40–82) | 20 (10–45) | | | 60 (45–73) | | 24 (10–40) |
| Number of infusion sites/infusion, median (IQR) | 4 (2–4) | 2 (2–2) | 4 (4–4) | | 2 (2–2) | | 3 (2–3) | 2 (1–2) | | | 2 (2–3) | | 2 (1–2) |
| 1 site, n (%) | 0 | 2 (15.4) | 0 | | 1 (11.1) | | 1 (2.0) | 9 (32.1) | | | 1 (1.7) | | 9 (33.3) |
| 2 sites, n (%) | 2 (33.3) | 9 (69.2) | 1 (20.0) | | 8 (88.9) | | 24 (47.1) | 18 (64.3) | | | 32 (53.3) | | 17 (63.0) |
| 3 sites, n (%) | 0 | 2 (15.4) | 0 | | 0 | | 15 (29.4) | 0 | | | 16 (26.7) | | 0 |
| >3 sites, n (%) | 4 (66.7) | 0 | 4 (80.0) | | 0 | | 11 (21.6) | 1 (3.6) | | | 11 (18.3) | | 1 (3.7) |
| Number of infusions/month/patient, median (IQR) | 4 (2–4) | 4 (4–8) | 4 (2–4) | | 4 (4–4) | | 4 (4–4) | 4 (4–8) | | | 4 (4–4) | | 4 (4–8) |
| Maximal infusion rate/site, median (IQR), (ml/h)^c^ | 40.0  (35.0–45.0) | – | 50.9  (50.9–50.9) | | – | | 43.3  (32.7–58.0) | – | | | 40.0  (34.0–59.0) | | – |
| Infusions, n (%), that were: |  |  |  | |  | |  |  | | |  | |  |
| Interrupted | – | 0 | – | | 0 | | – | 0 | | | – | | 1 (2.4) |
| Slowed | 0 | – | 0 | | – | | 1 (1.9) | – | | | 1 (1.5) | | – |
| Neither slowed nor interrupted | 5 (83.3) | 7 (41.2) | 4 (80.0) | | 8 (66.7) | | 49 (92.5) | 23 (57.5) | | | 59 (90.8) | | 17 (40.5) |
| Unknown | 1 (16.7) | 10 (58.8) | 1 (20.0) | | 4 (33.3) | | 3 (5.7) | 17 (42.5) | | | 5 (7.7) | | 24 (57.1) |
| **Dosing parameters** | | | | | | | | | | | | | |
| Weekly dose, median (IQR), g | 8.0  (7.0–10.0) | 6.5  (6.0–8.0) | 7.0  (5.8–11.0) | 6.5  (5.0–7.5) | | 8.0  (6.0–12.0) | | | 8.0  (6.0–8.0) | 8.0  (6.0–10.0) | | 8.0  (6.0–8.0) | |
| Weekly dose per kg, median (IQR), g/kg | 0.1 (0.1–0.1) | 0.1 (0.1–0.1) | 0.1 (0.1–0.1) | 0.1 (0.1–0.1) | | 0.1 (0.1–0.1) | | | 0.1 (0.1–0.1) | 0.1 (0.1–0.1) | | 0.1 (0.1–0.2) | |
| Dosing interval, n (%) |  |  |  |  | |  | | |  |  | |  | |
| Daily | 0 | – | 0 | – | | 1 (1.9) | | | – | 0 | | – | |
| 2–6 times/week | 0 | 5 (29.4) | 0 | 2 (16.7) | | 1 (1.9) | | | 18 (45.0) | 2 (3.1) | | 17 (40.5) | |
| Once weekly | 4 (66.7) | 12 (70.6) | 3 (60.0) | 10 (83.3) | | 43 (81.1) | | | 22 (55.0) | 56 (87.5) | | 25 (59.5) | |
| Every 2 weeks | 2 (33.3) | – | 2 (40.0) | – | | 7 (13.2) | | | – | 4 (6.3) | | – | |
| Other | 0 | – | 0 | – | | 1 (1.9) | | | – | 2 (3.1) | | – | |
| IgG trough levels, median (IQR), g/l^d^ | 6.7  (3.4–9.1) | 8.2  (7.8–11.4) | 4.7  (4.7–4.7) | 11.6  (11.5–11.6) | | 9.7  (8.0–10.7) | | | 8.7  (6.8–11.6) | 8.6  (7.9–10.6) | | 9.1  (8.3–11.0) | |

^a^ Cohort 1 only.

^b^ Cohorts 1 and 2 only.

^c^ Maximal infusion rate per site can only be analyzed for patients using pump administration and is the same for all sites in that infusion.

^d^ Available data: initiation, n=4 (infusion pump), n=5 (manual); 3 months, n=1 (infusion pump), n=2 (manual); 6 months, n=36 (infusion pump), n=10 (manual); 12 months, n=39 (infusion pump), n=20 (manual).

## Table S3. HRQoL assessments at 12 months post-initiation by mode of administration and indication

| **HRQoL assessments**^a^ | **Mode of administration** | | **Indication for treatment** | |
| --- | --- | --- | --- | --- |
|  | **Infusion pump**  **(n=71)** | **Manual**  **(n=54)** | **PID**  **(n=61)** | **SID**  **(n=64)** |
| **TSQM-9 score, mean (SD)** | | | | |
| Global satisfaction | 80.9 (20.0) | 77.1 (19.4) | 79.3 (23.2) | 79.5 (16.3) |
| Effectiveness | 76.8 (21.3) | 79.9 (17.6) | 75.9 (24.5) | 79.8 (14.5) |
| Convenience | 76.2 (16.5) | 73.5 (14.8) | 76.0 (17.0) | 74.4 (14.9) |
| **LQI score, mean (SD)** | | | | |
| Treatment interferences | 91.8 (9.7) | 93.4 (7.8) | 91.4 (10.3) | 93.3 (7.7) |
| Therapy-related problems | 85.3 (15.1) | 84.8 (10.6) | 82.7 (16.2) | 87.4 (9.6) |
| Therapy setting | 95.6 (7.6) | 95.4 (8.1) | 95.6 (7.6) | 95.3 (8.0) |
| Treatment costs | 91.6 (12.3) | 94.3 (9.2) | 93.3 (10.4) | 92.1 (12.0) |
| **TPQ score, n (%)** | | | | |
| Where did you receive your IG therapy before you participated in this study? |  |  |  |  |
| At the hospital | 16 (25.8) | 11 (28.2) | 13 (27.1) | 14 (26.4) |
| At the doctor’s office | 3 (4.8) | 1 (2.6) | 2 (4.2) | 2 (3.8) |
| At home | 47 (75.8) | 28 (71.8) | 36 (75.0) | 39 (73.6) |
| Other | 0 | 1 (2.6) | 0 | 1 (1.9) |
| Where do you prefer to receive your IG therapy? |  |  |  |  |
| At the hospital | 1 (1.6) | 0 | 0 | 1 (1.9) |
| At the doctor’s office | 1 (1.6) | 1 (2.6) | 1 (2.1) | 1 (1.9) |
| Other | 59 (95.2) | 38 (97.4) | 47 (97.9) | 50 (94.3) |
| At home | 1 (1.6) | 0 | 0 | 1 (1.9) |
| Extent to which you like or dislike the following aspects of Ig20Gly: |  |  |  |  |
| Frequency of administration |  |  |  |  |
| I like it very much | 15 (24.2) | 4 (10.3) | 12 (25.0) | 7 (13.2) |
| I like it | 26 (41.9) | 21 (53.8) | 23 (47.9) | 24 (45.3) |
| No preference | 18 (29.0) | 12 (30.8) | 10 (20.8) | 20 (37.7) |
| I dislike it | 3 (4.8) | 2 (5.1) | 3 (6.3) | 2 (3.8) |
| I dislike it very much | 0 | 0 | 0 | 0 |
| Number of punctures per month |  |  |  |  |
| I like it very much | 7 (11.3) | 3 (7.7) | 7 (14.6) | 3 (5.7) |
| I like it | 19 (30.6) | 12 (30.8) | 11 (22.9) | 20 (37.7) |
| No preference | 27 (43.5) | 20 (51.3) | 22 (45.8) | 25 (47.2) |
| I dislike it | 9 (14.5) | 4 (10.3) | 8 (16.7) | 5 (9.4) |
| I dislike it very much | 0 | 0 | 0 | 0 |
| Total time required for my therapy per month |  |  |  |  |
| I like it very much | 10 (16.1) | 4 (10.3) | 8 (16.7) | 6 (11.3) |
| I like it | 23 (37.1) | 20 (51.3) | 22 (45.8) | 21 (39.6) |
| No preference | 24 (38.7) | 13 (33.3) | 12 (25.0) | 25 (47.2) |
| I dislike it | 5 (8.1) | 2 (5.1) | 6 (12.5) | 1 (1.9) |
| I dislike it very much | 0 | 0 | 0 | 0 |
| Ease of administration |  |  |  |  |
| I like it very much | 14 (22.6) | 9 (23.7) | 15 (31.9) | 8 (15.1) |
| I like it | 36 (58.1) | 14 (36.8) | 19 (40.4) | 31 (58.5) |
| No preference | 10 (16.1) | 13 (34.2) | 10 (21.3) | 13 (24.5) |
| I dislike it | 2 (3.2) | 2 (5.3) | 3 (6.4) | 1 (1.9) |
| I dislike it very much | 0 | 0 | 0 | 0 |
| Option of self-administration |  |  |  |  |
| I like it very much | 38 (61.3) | 28 (71.8) | 31 (64.6) | 35 (66.0) |
| I like it | 18 (29.0) | 10 (25.6) | 13 (27.1) | 15 (28.3) |
| No preference | 3 (4.8) | 1 (2.6) | 3 (6.3) | 1 (1.9) |
| I dislike it | 3 (4.8) | 0 | 1 (2.1) | 2 (3.8) |
| I dislike it very much | 0 | 0 | 0 | 0 |
| Option to adjust my treatment to my own schedule |  |  |  |  |
| I like it very much | 34 (54.8) | 27 (69.2) | 31 (64.6) | 30 (56.6) |
| I like it | 26 (41.9) | 9 (23.1) | 15 (31.3) | 20 (37.7) |
| No preference | 2 (3.2) | 2 (5.1) | 1 (2.1) | 3 (5.7) |
| I dislike it | 0 | 1 (2.6) | 1 (2.1) | 0 |
| I dislike it very much | 0 | 0 | 0 | 0 |
| Convenience in general |  |  |  |  |
| I like it very much | 25 (40.3) | 18 (46.2) | 24 (50.0) | 19 (35.8) |
| I like it | 31 (50.0) | 20 (51.3) | 19 (39.6) | 32 (60.4) |
| No preference | 6 (9.7) | 1 (2.6) | 5 (10.4) | 2 (3.8) |
| I dislike it | 0 | 0 | 0 | 0 |
| I dislike it very much | 0 | 0 | 0 | 0 |
| Amount of time the administration requires |  |  |  |  |
| I like it very much | 12 (19.4) | 5 (12.8) | 12 (25.0) | 5 (9.4) |
| I like it | 31 (50.0) | 23 (59.0) | 24 (50.0) | 30 (56.6) |
| No preference | 12 (19.4) | 9 (23.1) | 5 (10.4) | 16 (30.2) |
| I dislike it | 6 (9.7) | 2 (5.1) | 6 (12.5) | 2 (3.8) |
| I dislike it very much | 1 (1.6) | 0 | 1 (2.1) | 0 |
| Complexity of the process of administration |  |  |  |  |
| I like it very much | 16 (26.2) | 10 (25.6) | 16 (34.0) | 10 (18.9) |
| I like it | 26 (42.6) | 16 (41.0) | 20 (42.6) | 22 (41.5) |
| No preference | 17 (27.9) | 11 (28.2) | 9 (19.1) | 19 (35.8) |
| I dislike it | 1 (1.6) | 2 (5.1) | 1 (2.1) | 2 (3.8) |
| I dislike it very much | 1 (1.6) | 0 | 1 (2.1) | 0 |
| Option of self-administration by myself, without medical supervision |  |  |  |  |
| I like it very much | 37 (59.7) | 26 (66.7) | 32 (66.7) | 31 (58.5) |
| I like it | 23 (37.1) | 12 (30.8) | 14 (29.2) | 21 (39.6) |
| No preference | 2 (3.2) | 1 (2.6) | 2 (4.2) | 1 (1.9) |
| I dislike it | 0 | 0 | 0 | 0 |
| I dislike it very much | 0 | 0 | 0 | 0 |
| As you are now experienced in receiving your IG therapy in a variety of ways, would you like to continue your treatment with Ig20Gly subcutaneously? |  |  |  |  |
| Yes | 62 (100) | 39 (100) | 48 (100) | 53 (100) |
| No | 0 | 0 | 0 | 0 |

^a^ Values are presented for those patients who provided feedback.

## Table S4. AEs by mode of Ig20Gly administration

|  | **AEs of interest** | | | | **Other AEs** | | | | **SAEs** | | | |
| --- | --- | --- | --- | --- | --- | --- | --- | --- | --- | --- | --- | --- |
|  | **Infusion pump** | | **Manual** | | **Infusion pump** | | **Manual** | | **Infusion pump** | | **Manual** | |
|  | **Patients n (%)**  **(N=71)** | **AEs,**  **n** | **Patients**  **n (%)**  **(N=54)** | **AEs,**  **n** | **Patients**  **n (%)**  **(N=71)** | **AEs,**  **n** | **Patients**  **n (%)**  **(N=54)** | **AEs,**  **n** | **Patients**  **n (%)**  **(N=71)** | **AEs,**  **n** | **Patients**  **n (%)**  **(N=54)** | **AEs,**  **n** |
| **Any AE** | 6 (8.5) | 9 | 9 (16.7) | 11 | 12 (16.9) | 13 | 8 (14.8) | 16 | 5 (7.0) | 5 | 0 | 0 |
| **Severity of AE** |  |  |  |  |  |  |  |  |  |  |  |  |
| Mild | 4 (5.6) | 7 | 6 (11.1) | 6 | 6 (8.5) | 7 | 5 (9.3) | 7 | 0 | 0 | 0 | 0 |
| Moderate | 1 (1.4) | 1 | 4 (7.4) | 5 | 3 (4.2) | 3 | 3 (5.6) | 9 | 1 (1.4) | 1 | 0 | 0 |
| Severe | 1 (1.4) | 1 | 0 | 0 | 3 (4.2) | 3 | 0 | 0 | 4 (5.6) | 4 | 0 | 0 |
| **AEs considered related to Ig20Gly** |  |  |  |  |  |  |  |  |  |  |  |  |
| Related | 3 (4.2) | 3 | 4 (7.4) | 5 | 2 (2.8) | 3 | 1 (1.9) | 1 | 0 | 0 | 0 | 0 |
| Possibly related | 2 (2.8) | 2 | 3 (5.6) | 3 | 2 (2.8) | 2 | 2 (3.7) | 4 | 1 (1.4) | 1 | 0 | 0 |
| Probably related | 1 (1.4) | 1 | 1 (1.9) | 1 | 0 | 0 | 2 (3.7) | 2 | 0 | 0 | 0 | 0 |

## Table S5. Infusion and dosing parameters by indication

|  | **Initiation**^a^ | | **Post-initiation** | | | | | |
| --- | --- | --- | --- | --- | --- | --- | --- | --- |
|  |  |  | **3 months**^a^ | | **6 months**^b^ | | **12 months** | |
|  | **PID**  **(n=10)** | **SID**  **(n=13)** | **PID**  **(n=7)** | **SID**  **(n=10)** | **PID**  **(n=40)** | **SID**  **(n=53)** | **PID**  **(n=52)** | **SID**  **(n=55)** |
| **Infusion parameters** | | | | | | | | |
| Infusion volume/infusion, median (IQR), ml | 35 (30–40) | 30 (20–40) | 40 (30–50) | 30 (20–40) | 40 (25–60) | 40 (30–50) | 40 (25–50) | 40 (35–50) |
| Infusion duration, median (IQR), minutes | 30 (15–60) | 40 (15–55) | 15 (10–60) | 37 (15–45) | 43 (15–65) | 45 (30–60) | 43 (20–65) | 60 (40–65) |
| Number of infusion sites/infusion, median (IQR) | 2 (2–3) | 2 (2–3) | 2 (2–4) | 2 (2–4) | 2 (2–4) | 2 (2–3) | 2 (2–4) | 2 (2–3) |
| 1 site, n (%) | 1 (11.1) | 1 (10.0) | 1 (14.3) | 0 | 7 (21.9) | 3 (6.4) | 8 (18.6) | 2 (4.5) |
| 2 sites, n (%) | 5 (55.6) | 6 (60.0) | 4 (57.1) | 5 (71.4) | 14 (43.8) | 28 (59.6) | 21 (48.8) | 28 (63.6) |
| 3 sites, n (%) | 1 (11.1) | 1 (10.0) | 0 | 0 | 3 (9.4) | 12 (25.5) | 3 (7.0) | 13 (29.5) |
| >3 sites, n (%) | 2 (22.2) | 2 (20.0) | 2 (28.6) | 2 (28.6) | 8 (25.0) | 4 (8.5) | 11 (25.6) | 1 (2.3) |
| Number of infusions/month/patient, median (IQR) | 4 (4–4) | 4 (4–8) | 4 (4–4) | 4 (4–4) | 4 (4–8) | 4 (4–4) | 4 (4–8) | 4 (4–4) |
| Maximal infusion rate/site, median (IQR) (ml/h)^c^ | 35.0  (35.0–35.0) | 45.0  (45.0–45.0) | N/A | 50.9  (50.9–50.9) | 34.0  (28.0–40.0) | 45.0  (32.7–60.0) | 33.5  (25.7–40.0) | 40.0  (35.0–60.0) |
| Infusions, n (%), that were: |  |  |  |  |  |  |  |  |
| Interrupted | – | 0 | – | 0 | – | 0 | – | 1 (1.8) |
| Slowed | 0 | – | 0 | – | 1 (2.5) | – | 1 (1.9) | – |
| Neither slowed nor interrupted | 5 (50.0) | 7 (53.8) | 4 (57.1) | 8 (80.0) | 27 (67.5) | 45 (84.9) | 37 (71.2) | 39 (70.9) |
| Unknown | 5 (50.0) | 6 (46.2) | 3 (42.9) | 2 (20.0) | 12 (30.0) | 8 (15.1) | 14 (26.9) | 15 (27.3) |
| **Dosing parameters** | | | | | | | | |
| Weekly dose, median (IQR), g | 8.0 (6.0–8.0) | 7.0 (6.0–8.0) | 5.8 (5.5–6.0) | 7.5 (6.0–8.0) | 8.0 (6.0–12.0) | 8.0 (6.0–10.0) | 9.0 (7.0–12.0) | 8.0 (6.0–10.0) |
| Weekly dose per kg, median (IQR), g/kg | 0.1 (0.1–0.1) | 0.1 (0.1–0.1) | 0.1 (0.1–0.1) | 0.1 (0.1–0.1) | 0.1 (0.1–0.2) | 0.1 (0.1–0.1) | 0.1 (0.1–0.2) | 0.1 (0.1–0.1) |
| Dosing interval, n (%) |  |  |  |  |  |  |  |  |
| Daily | – | 0 | – | 0 | – | 1 (1.9) | – | 0 |
| 2–6 times/week | 0 | 5 (38.5) | 0 | 2 (20.0) | 12 (30.0) | 7 (13.2) | 15 (28.8) | 4 (7.4) |
| Once weekly | 9 (90.0) | 7 (53.8) | 6 (85.7) | 7 (70.0) | 25 (62.5) | 40 (75.5) | 34 (65.4) | 47 (87.0) |
| Every 2 weeks | 1 (10.0) | 1 (7.7) | 1 (14.3) | 1 (10.0) | 2 (5.0) | 5 (9.4) | 2 (3.8) | 2 (3.7) |
| Other | 0 | 0 | 0 | 0 | 1 (2.5) | 0 | 1 (1.9) | 1 (1.9) |
| IgG trough levels, median (IQR), g/l^d^ | 7.7  (6.0–11.4) | 8.2  (7.4–10.7) | – | 11.5  (4.7–11.6) | 10.4  (9.3–11.8) | 8.4  (7.8–10.0) | 9.9  (8.6–11.8) | 8.3  (7.9–9.0) |

^a^ Cohort 1 only.

^b^ Cohorts 1 and 2 only.

^c^ Maximal infusion rate per site can only be analyzed for patients using pump administration and is the same for all sites in that infusion.

^d^ Available data: initiation, n=6 (PID), n=3 (SID); 3 months, n=3 (SID); 6 months, n=16 (PID), n=30 (SID); 12 months, n=28 (PID), n=31 (SID).

## Table S6. AEs by indication

| **Parameter,**  **n (%)** | **AEs of interest** | | | | **Other AEs** | | | | **SAEs** | | | |
| --- | --- | --- | --- | --- | --- | --- | --- | --- | --- | --- | --- | --- |
|  | **PID** | | **SID** | | **PID** | | **SID** | | **PID** | | **SID** | |
|  | **Patients**  **n (%)**  **(N=61)** | **AEs,**  **n** | **Patients**  **n (%)**  **(N=64)** | **AEs,**  **n** | **Patients**  **n (%)**  **(N=61)** | **AEs,**  **n** | **Patients**  **n (%)**  **(N=64)** | **AEs,**  **n** | **Patients**  **n (%)**  **(N=61)** | **AEs,**  **n** | **Patients**  **n (%)**  **(N=64)** | **AEs,**  **n** |
| **Any AE** | 10 (16.4) | 15 | 5 (7.8) | 5 | 8 (13.1) | 14 | 12 (18.8) | 15 | 1 (1.6) | 1 | 4 (6.3) | 4 |
| **Severity of AE** |  |  |  |  |  |  |  |  |  |  |  |  |
| Mild | 7 (11.5) | 10 | 3 (4.7) | 3 | 6 (9.8) | 8 | 5 (7.8) | 6 | 0 | 0 | 0 | 0 |
| Moderate | 3 (4.9) | 4 | 2 (3.1) | 2 | 2 (3.3) | 6 | 4 (6.3) | 6 | 0 | 0 | 1 (1.6) | 1 |
| Severe | 1 (1.6) | 1 | 0 | 0 | 0 | 0 | 3 (4.7) | 3 | 1 (1.6) | 1 | 3 (4.7) | 3 |
| **AEs considered related to Ig20Gly** |  |  |  |  |  |  |  |  |  |  |  |  |
| Related | 5 (8.2) | 6 | 2 (3.1) | 2 | 2 (3.3) | 2 | 1 (1.6) | 2 | 0 | 0 | 0 | 0 |
| Possibly related | 3 (4.9) | 3 | 2 (3.1) | 2 | 2 (3.3) | 2 | 2 (3.1) | 4 | 1 (1.6) | 1 | 0 | 0 |
| Probably related | 1 (1.6) | 1 | 1 (1.6) | 1 | 1 (1.6) | 1 | 1 (1.6) | 1 | 0 | 0 | 0 | 0 |
